# Control Strategy for Process Development of High-Shear Wet Granulation and Roller Compaction to Prepare a Combination Drug Using Integrated Quality by Design

**DOI:** 10.3390/pharmaceutics13010080

**Published:** 2021-01-08

**Authors:** Ji Yeon Kim, Myung Hee Chun, Du Hyung Choi

**Affiliations:** 1 Department of Pharmaceutical Engineering, Inje University, Gyeongnam 621-749, Korea; delayeon18@naver.com; 2 School of Pharmacy, Sungkyunkwan University, Suwon 16419, Korea; hiyachun@naver.com

**Keywords:** quality by design, multivariate analysis, control strategy, intermediate quality attributes, high-shear wet granulation, roller compaction

## Abstract

In this study, we developed a control strategy for a drug product prepared by high-shear wet granulation and roller compaction using integrated quality by design (QbD). During the first and second stages, we optimized the process parameters through the design of experiments and identified the intermediate quality attributes (IQAs) and critical quality attributes (CQAs) relationship, respectively. In the first stage, we conducted an initial risk assessment by selecting critical process parameters with high impact on IQAs and CQAs and confirmed the correlation between control and response factors. Additionally, we performed Monte Carlo simulations by optimizing the process parameters to deriving and building a robust design space. In the second stage, we identified the IQAs and CQAs relationship for the control strategy, using multivariate analysis (MVA). Based on MVA, in the metformin layer, dissolution at 1 h was significantly correlated with intrinsic dissolution rate and granule size, and dissolution at 3 h was significantly correlated with bulk density and granule size. In dapagliflozin layer, dissolution at 10 min and 15 min was significantly correlated with granule size. Our results suggest that the desired drug quality may result through IQAs monitoring during the process and that the integrated QbD approach utilizing MVA can be used to develop a control strategy for producing high-quality drug products.

## 1. Introduction

The drug manufacturing process is initially developed at the laboratory scale; it is then scaled up to the pilot scale and the commercial scale. In the same manner, a design space for process parameters is prepared by the quality by design (QbD) approach at the laboratory scale. However, it is difficult to apply a design space built at the laboratory scale to the pilot or commercial scale because of process variability parameters such as batch size variability, changes in the process parameter values, and changes in the manufacturing equipment during scale-up [1,2,3]. Therefore, a scale-up strategy is essential for the QbD approach. In general, model-based scale-up strategies such as empirical models, physics-based models, and engineering principle-based models have been used in the pharmaceutical industry [4]. The empirical models are used to identify the relationship between input (control factors) and output variables (response factors) [5]. Empirical models include multivariate analysis (MVA), design of experiment (DoE), and process analytical technology (PAT) based on empirical, semi-empirical, or statistical methods [6]. Physics-based models include the discrete element model, computational fluid dynamics, the finite element method, and hybrid models [5]. Engineering principles-based models include the dimensionless number, thermodynamic model, and heat and mass transfer model [7]. 

QbD in pharmaceutical development focuses on process design, understanding the relationship between control factors [critical material attributes (CMAs) and critical process parameters (CPPs)] and response factors [intermediate quality attributes (IQAs) and critical quality attributes (CQAs)], and control CPPs [3,8]. Pharmaceutical manufacturing processes must be robustly designed and fully understood. Rigidly designed manufacturing processes and a full understanding of the process provide regulatory and economic advantages [9]. In general, understanding a process means that all variables are identified and described, variability is controlled by the process, and product quality attributes can be accurately and reliably predicted. Sufficient information needs to be gathered to clearly understand these relationships. DoE can be a powerful tool for collecting sufficient information and a powerful statistical technique used to study the effects of various variables affecting the drug product. The use of DoE increases the understanding of the process and provides insight, leading to process improvement and cost reduction [4]. 

Active pharmaceutical ingredient (API) variability, excipient variability, and process variability affect the drug product quality [8,10]. In the manufacturing process development stage, API variability and excipient variability might have a low effect on the quality of drug products because fixed values such as drug product formulation and API specifications are used at the pilot scale or the commercial scale. However, process variability (e.g., values of process variables and types of equipment used in the process) can have a high impact on the quality of the drug product. The manufacturing process consists of unit operations to produce the desired quality product. Pharmaceutical unit operations include mixing, granulation, milling, drying, compression, and coating. After a unit operation is completed, the intermediate product, which is a substance, undergoes physical and chemical changes. In particular, the physical properties of granules produced through granulation are greatly influenced by process variables, which can seriously affect the quality of the drug product. The pharmaceutical granulation process entails wet and dry granulation. Wet granulation involves low-shear, high-shear, and fluid bed. There are two dry granulation methods; slugging and roller compaction. In high-shear wet granulation process variability affects drug product quality. For example, inadequate impeller speed leads to inadequate granule growth [11,12,13]. Inadequate granule growth leads to unwanted granular porosity and strength, which in turn affects the drug product. In roller compaction the pressure of the roller is an important parameter determining powder cohesion [14]. Powder cohesion is directly related to ribbon density [14]. As the pressure of the roller increases, the force exerted on the powder increases, thereby increasing the strength of the ribbon [15]. The roller gap also affects the physical properties of the ribbon. Even if the same force is applied to the ribbon, the degree of transmission of the force varies depending on the thickness of the ribbon. If the ribbon is thick, its strength decreases, which, in turn, results in smaller and weaker granules [16]. In the granulation process, the variability in the design space occurs due to the variability of process variables, so a scale-up strategy is required accordingly. 

In order to use the model-based strategy mentioned above, it is necessary to secure correlations between process parameters, intermediate products, and drug products as well as to select and manage CPPs, IQAs, and CQAs. The relationship between CPPs and IQAs and the relationship between CPPs and CQAs can be identified through the DoE process, but the relationship between IQAs and CQAs is difficult to identify. Therefore, the DoE process should be supplemented. MVA can be used to analyze multiple variables simultaneously, and can act complementary to the DoE process because it is useful for the intensive management of process parameters or material properties. In fact, many studies have analyzed the correlation between intermediate products and drug products using MVA. Huang et al. used MVA methods such as PCA and PLS to identify the correlation between control factors and response factors and the correlation among response factors [17]. As a result, CPPs, such as compression force, wet massing time, and water amount, and IQAs, such as particle size and loss on drying (LOD), were significantly correlated with dissolution. In addition, Haware, et al. used PCA and PLS to identify the correlation between control factors and response factors and the correlation among response factors [18]. As a result, PCA identified that the Hausner ratio, work of compression, and tensile strength are negatively correlated with the yield pressure of plastic and elastic deformation. Using empirical models such as DoE and MVA, correlations between control factors and response factors and correlations between response factors can be identified. A scale-up strategy can be secured by managing and controlling CPPs and IQAs based on these relationships and by monitoring the correlations through techniques such as PAT.

The objective of this study was to develop a control strategy for a combination drug prepared by the high-shear wet granulation and roller compaction processes using the integrated QbD approach. The metformin and dapagliflozin might show a poor compaction property, it is difficult to produce tablets which have acceptable mechanical strength [19]. This disadvantage can overcome using high-shear wet granulation and roller compaction. The response surface design was used to obtain optimal the process parameters for each process. Based on the initial risk assessment CPPs, IQAs, and CQAs were selected as control factors and response factors for DoE. The effect of CPPs on IQAs and CQAs was analyzed using coded equations. A design space was established to satisfy the target values using coded equations. Monte Carlo simulations were conducted to evaluate the risk of uncertainty in design space predictions. In addition, by utilizing MVA methods, such as the Pearson correlation coefficient and PCA, we confirmed the relationships between IQAs and CQAs. 

## 2. Materials and Methods

### 2.1. Materials

Metformin and dapagliflozin were supplied from Kyung-dong pharm (Seoul, Korea). Hydroxypropyl methylcellulose (100,000 cps, SR type) and Low-substituted hydroxypropyl cellulose (L-HPC) were purchased from Shin-Etsu Chemical Co., Ltd. (Tokyo, Japan). Lactose monohydrate, magnesium stearate, silicon dioxide, and microcrystalline cellulose were purchased from Sigma-Aldrich Co. (St. Louis, MO, USA). Calcium silicate was purchased from Fluka (Buchs, Switzerland). All other reagents were analytical or HPLC grade.

### 2.2. Experimental Design to Optimize the Manufacturing Process

#### 2.2.1. Experimental Design of High-Shear Wet Granulation

The experimental design used to optimize the manufacturing process of high-shear wet granulation was produced using Design-Expert^®^ software (version 12; Stat-Ease Inc., Minneapolis, MN, USA). Response surface design was used to identify the optimal process parameters of high-shear wet granulation with three control factors: *p*_1_ (impeller speed), *p*_2_ (massing time), and *p*_3_ (binder solvent). The control factors were in the 50–100 rpm, 10–50 s, and 1–4 mL ranges, respectively. IQAs, such as intrinsic dissolution rate (*q*_1_), granule size (*q*_2_–*q*_6_), true density (*q*_7_), bulk density (*q*_8_), Carr’s index (*q*_9_), angle of repose (*q*_10_), and granule strength (*q*_11_), and CQAs, such as swelling property at 1 h (*q*_12_), 3 h (*q*_13_), and 5 h (*q*_14_); weight gain at 1 h (*q*_15_), 3 h (*q*_16_), and 5 h (*q*_17_); mass loss at 1 h (*q*_18_), 3 h (*q*_19_), and 5 h (*q*_20_); gel strength at 1 h (*q*_21_), 3 h (*q*_22_), and 5 h (*q*_23_); dissolution at 1 h (*q*_24_), 3 h (*q*_25_), and 10 h (*q*_26_); and contact angle (*q*_27_), were evaluated as response factors.

The test drug components used were metformin (API) 1000 mg, calcium silicate (excipient) 30 mg, HPMC (binder) 10 mg, HPMC (release control agent) 255 mg, and St-Mg (lubricant) 10 mg. To prepare the test drug, metformin granules were using a high-shear granulator (MIXER TORQUE RHEOMETER 3; Caleva, Sturminster Newton, UK). Purified water was selected as the binding solvent. The process parameters (impeller speed, massing time, and binder solvent) were set following the experimental design, and the binding solvent spray speed was fixed at 100 mL/h. After granulation, the mass granules were dried in an oven at 50 °C for 1 h. Dry granules that reached the appropriate water content were sieved through a #25-mesh sieve to remove any aggregates. IQAs were evaluated using intermediate products after granulation (dried granules). HPMC and St-Mg were added to the dried granules and mixed using an LM 40 Lab Blender (L.B. Bohle, Ennigerloh, Germany).

#### 2.2.2. Experimental Design of Roller Compaction

To optimize the manufacturing process of roller compaction, the experimental design was produced using Design-Expert^®^ software (version 12; Stat-Ease Inc., Minneapolis, MN, USA). Response surface design was used to identify the optimal process parameters of roller compaction with three control factors: *c*_1_ (roller pressure), *c*_2_ (roller gap), and *c*_3_ (mill screen size). The control factors were in the 25–85 bar, 1.2–2.4 mm, and 0.5–1.5 mm ranges, respectively. IQAs, such as the intrinsic dissolution rate (*d*_1_), granule size (*d*_2_–*d*_6_), ribbon density (*d*_7_), bulk density (*d*_8_), tapped density (*d*_9_), granule strength (*d*_10_), and granule uniformity (*d*_11_), and CQAs, such as tablet C.U. (*d*_12_); dissolution at 5 min (*d*_13_), 10 min (*d*_14_), and 15 min (*d*_15_); and contact angle (*d*_16_), were evaluated as response factors. 

The test drug components used were dapagliflozin (API) 15.63 mg, MCC (excipient) 191.37 mg, lactose (excipient) 10.00 mg, L-HPC (disintegrants) 20.00 mg, silicon dioxide 8.00 mg, and St-Mg (lubricant) 2.00 mg and 3.00 mg (post-mix). To prepare the test drug, dapagliflozin granules were produced using a POLYGRAN^®^ (Gerteis, Rapperswil-Jana, Switzerland). The process parameters (roller pressure, roller gap, and mill screen size) were set following the experimental design, and roll speed, feed screw, and roller pressure were fixed at 4 rpm, 10 rpm, and 55 bar, respectively. The ribbon was formed by the force of the compression roller from the powder mixture transferred by the screw feeder. The formed ribbon was crushed into small particles to form dry granules. After granulation, St-Mg was added to the intermediate product, which was then mixed using an LM 40 Lab Blender (Bohle, Germany).

### 2.3. Measurement of IQAs and CQAs

#### 2.3.1. Measurement of Intrinsic Dissolution Rate

The intrinsic dissolution test used two methods. The first method is USP <1087> stationary disk apparatus for metformin granules [20]. Metformin granules content HPMC which has swelling property. Due to the property of HPMC, it is difficult to test the drug release behavior at a constant surface area in the Franz diffusion cell, so metformin granules were tested using a different method. After weighing 300 mg of metformin granules, they were put into a die and then compressed with a pressure of 200 kg/cm^2^ using a single punch press (RIKEN KIKI Co., Ltd., Tokyo, Japan) to produce a drug disk. One surface of the drug disk was exposed, and the surface was covered with a membrane filter (cellulose acetate, 0.2 µm) [21]. Then, the die was placed on the bottom of the vessel with a flat bottom (Distek Inc., North Brunswick, Nj, USA). In the intrinsic dissolution test, 1000 mL of pH 6.8 phosphate buffer maintained at 37 ± 0.5 °C was used as the dissolution medium, and the paddle speed was 100 rpm. At the sampling time, 5 mL of sample was withdrawn and filtered through a 0.45 µm membrane filter to remove impurities. The intrinsic dissolution rate was calculated from Equation (1) [22].

The second method is Franz diffusion cell for dapagliflozin granules. The test was conducted by Franz diffusion cell tester (Logan instruments Corp., Somerset, NJ, USA). Before testing, the membrane filter (cellulose acetate, 0.2 µm) was wetted with the test medium. After wetting membrane filter was put on the tester, 200 mg of dapagliflozin granules were put into the test and spread flat to contact the membrane. As the test medium, a pH 6.8 phosphate buffer maintained at 37 ± 0.5 °C was used. The test was conducted for 3 h, and 5 mL of sample was withdrawn every 30 min, and then filtered through a 0.45 µm membrane filter.

(1) $$J\;=\;\frac{Vdc}{dt}\times \frac{1}{A}$$

where *J* is dissolution flow (µg mm^−2^ min^−1^), *V* is the volume of the dissolution medium (mL), *c* is the concentration of dissolved drug in the medium (µg/mL), *A* is surface area of the sample (mm^2^), and *t* is time (min).

#### 2.3.2. Measurement of Granule Properties (Granule Size, Granule Density, Flowability, and Granule Strength)

The laser diffraction method was used to evaluate the granule size. Approximately, a 5 g granule was introduced into a Malvern Mastersizer 3000E (Malvern Instruments Ltd., Worcestershire, UK). The granule size distribution diameters (D_10_, D_50_, D_90_, D(3,2), and D(4,3)) were determined. The measurements were repeated three times and the mean value was used in this study.

The granule density was evaluated as true density, bulk density, and tapped density. To evaluated granule true density, a helium pycnometer (AccuPyc 1330; Micromeritics Instrument Co., Norcross, GA, USA) was used. After accurately weighing the granules, the granules were poured into a sample cell, and then helium gas was filled into the sample cell to measure the pressure in the cell, thereby calculating the volume of the granules. Bulk density and tapped density were measured using a 10 mL mass cylinder. First, the bulk density was measured by pouring excess granules into a 10 mL mass cylinder and then scraping the top of the cylinder to remove excess granules. The tapped density was calculated by measuring the reduced volume by pouring the excess granules into a 10 mL mass cylinder, scraping the top of the cylinder to remove the excess granules, and then tapping the cylinder 100 times at a constant speed and height.

To evaluated granule flowability, Carr’s index and angle of repose was measured. The Carr’s index calculated using Equation (2). Granules fed through a funnel with a diameter of 12 mm until the powder formed a cone. Then, the angle of repose was measured by measuring the angle of the formed cone [23].

(2) $${\mathrm{Carr}}^{\text{’}}s\;\mathrm{index}\;=\;\frac{\rho_{T}-\rho_{B}}{\rho_{B}}\times 100\%$$

where ρ*_B_* is the bulk density of the granules and ρ*_T_* is the tapped density of granules.

The granule strength test was conducted by a texture analyzer (TA.XT plus, Stable Micro Systems Ltd., Surrey, UK). 710–850 µm granules were selected to test. 30 mg granules were accurately weighed and placed under the probe. Individual granule was compressed with a 10 mm cylinder probe. The test mode was operated in compression mode. The trigger force was set as 0.0049 N. Using area under the curve in the force versus distance graph measured granule strength.

For the test of granule strength, 30 mg of 710–850 µm granules were weighed and placed under the probe of the texture analyzer. The test used a texture analyzer (TA.XT plus, Stable Micro Systems Ltd., UK). The probe used a 10 mm cylinder probe and the test mode was operated in compression mode. The trigger force was set to 0.0049 N. Granule strength was measured by calculating the area under the curve in the force versus distance graph [24].

#### 2.3.3. Measurement of Swelling Property

To evaluated swelling of the metformin tablet swelling property test was conducted. The test tablet was prepared by weighing 1305 mg of metformin granules and insert granules in a 15 mm cylindrical-shaped die and compressed at 200 kg/cm^2^ using a single punch press (RIKEN SEIKI Co., Ltd., Japan). The front and back of the tablet were closed with clear acrylic plates (6 × 4 cm), and both sides were firmly held with a rubber band. Since both sides of the acrylic plates are exposed, when the acrylic plates are placed in the test medium, the medium enters the acrylic plates, and the tablet starts to swell as the tablet and the medium contact. The tablets fixed on the acrylic plate were immersed in 250 mL of pH 6.8 phosphate buffer, and a magnetic bar was placed on the acrylic plate, followed by stirring at 250 rpm using a magnetic stirrer (Scilab Korea Co., Ltd., Seoul, Korea). At a predetermined time, the acrylic plate was taken out and the diameters of the gelled and non-gelled portions of the tablet were measured using a digital caliper (Mitutoyo, Kawasaki-shi, Kanagawa, Japan). The swelling property was calculated using Equation (3) [25].

(3) $$\mathrm{Swelling}\;\mathrm{property}\;\left(\%\right)\;=\;\left\{1-\frac{{\left(L_{2}\right)}^{3}}{{\left(L_{1}\right)}^{3}}\right\}\;\times \;100$$

where *L*_2_ is diameter of the portion not gelled after the test and *L*_1_ is diameter of the tablet before the test.

#### 2.3.4. Measurement of Weight Gain and Mass Loss

To prepared the test tablets for weight gain and mass loss, 1305 mg of granules weighted and insert in a 15 mm cylindrical-shaped die and compressed at 200 kg/cm^2^ using a single punch press (RIKEN SEIKI Co., Ltd., Japan). To evaluated weight gain, 12 tablets were added to the 500 mL of pH 6.8 phosphate buffer, and then a magnetic bar was added and stirred at 450 rpm using a magnetic stirrer (Scilab Korea Co., Ltd., Korea). At a predetermined time, 4 tablets withdrawn from the medium, and the excess medium on the surface was removed using an absorbent tissue, and the weight of tablets was measured. After that, the swollen tablets were completely dried in an oven at 50 °C for mass loss testing and the dried tablets were weighted. The weight gain and mass loss were calculated using Equations (4)–(5), respectively [25].

(4) $$\mathrm{Weight}\;\mathrm{gain}\;\left(\%\right)\;=\;\frac{W_{2}-W_{1}}{W_{1}}\times 100$$

(5) $$\mathrm{Mass}\;\mathrm{loss}\;\left(\%\right)\;=\;\frac{W_{1}-W_{3}}{W_{1}}\times 100$$

where *W*_1_ is initial weight of tablet, *W*_2_, and *W*_3_ is weight of the tablet with water at time *t* and weight of the dried tablet, respectively.

#### 2.3.5. Measurement of Gel Strength

To prepare the test tablets for gel strength, 1305 mg of granules weighted and insert granules into a 15 mm semi-circular die and compressed at 200 kg/cm^2^ using a single punch press (RIKEN SEIKI Co., Ltd., Japan). Four tablets were put between two clear acrylic plates, and both ends were firmly fixed with a rubber band. Since both sides of the acrylic plates are exposed, when the acrylic plates are placed in the test medium, the medium enters the acrylic plates, and the tablet starts to swell as the tablet and the medium contact. The tablets fixed on the clear acrylic plate were immersed in 250 mL of pH 6.8 phosphate buffer, and a magnetic bar was placed on the acrylic plate, and then stirred at 250 rpm using a magnetic stirrer (Scilab Korea Co., Ltd., Korea). At a predetermined time, each tablet was withdrawn. The gel strength test was conducted by texture analyzer (TA.XT plus, Stable Micro Systems Ltd., UK). The probe used a 5 mm cylinder probe. The swollen tablet placed under the probe. The test mode was operated in compression mode, where the probe penetrated the gel layer at a speed of 1 mm/s. The gel strength was calculated using the area under the curve in force versus time.

#### 2.3.6. In Vitro Dissolution Test

To prepare the test tablets for metformin, 1305 mg of granules weighted and insert in a 15 mm cylindrical-shaped die and compressed at 200 kg/cm^2^ using a single punch press (RIKEN SEIKI Co., Ltd., Japan). To prepare the test tablets for dapagliflozin, 250 mg of granules weighted and insert in an 8 mm cylindrical-shaped die and compressed at 160 kg/cm^2^ using a single punch press (RIKEN SEIKI Co., Ltd., Japan). To prevent the tablet adhesion to the bottom of the vessel, dissolution was conducted according to the USP Apparatus 1 guidelines (Basket Apparatus) (ERWEKA GmbH, Langen, Germany) [26]. The dissolution medium used 1000 mL of pH 6.8 phosphate buffer maintained at 37 ± 0.5 °C. A basket rotation speed was set at 100 rpm. At a predetermined time, a 5 mL sample was withdrawn and filtered through a 0.45 µm membrane filter. After withdrawn the sample, the metformin sample diluted 20 times with the dissolution medium.

#### 2.3.7. Measurement of Contact Angle

The test medium was used dissolution medium (pH 6.8 phosphate buffer). Dropped 8 µL medium on a tablet and then measured contact angle imaging it with a video camera (Contact angle analyzer, Phoenix 300 TOUCH, SEO, Suwon-si, Korea). The angles were calculated directly from the video monitor. The contact angle was calculated Young’s equation using Equation (6) [27]. In order to obtain the rate at which water permeates into the tablet, used the slope of the time versus contact angle graph.

(6) $$\gamma_{SV}-\gamma_{SL}-\gamma_{LY}\cos\theta \;=\;0$$

where *γ_SV_*, *γ_SL_*, and *γ_LV_* are solid-vapor interfacial energy, solid-liquid interfacial energy, and liquid-vapor interfacial energy, respectively.

### 2.4. HPLC Analysis Method

To evaluated drug content HPLC analysis was conducted by HPLC (Agilent, Santa Clara, CA, USA). The method of HPLC analysis following: UV wavelength 255 nm for metformin and 224 nm for dapagliflozin, column used were an XTerra^®^ RP 18 (4.6 × 150 mm, 5 µm) (Waters, Santa Milford, MA, USA) maintained 40 °C, mobile phase was used 60:40 volume mixture of buffer (made by dissolving monoammonium phosphate and sodium dodecyl sulfate) and acetonitrile, flow rate was 1.5 mL/min, and the injection volume was 10 µL for metformin and 20 µL for dapagliflozin. To prepare a stock standard solution, accurately 12.00 mg of metformin and 18.756 mg of dapagliflozin were inserted into a 10 mL of clean dry volumetric flasks add about 7 mL of methanol and sonicate to dissolve and removal of air completely and make volume up to the mark with the same methanol. To prepared calibration standards the metformin stock solution was diluted 20 times and dapagliflozin stock solution was diluted 100 times. From this solution calibration standards, ranging from 60.00 to 5.00 µg/mL of metformin and from 18.756 to 1.563 µg/mL of dapagliflozin, were prepared before quantitative analysis.

### 2.5. Multivariate Analysis

MVA methods, such as the Pearson correlation coefficient and PCA, were used to identify the correlation between IQAs and CQAs. PCA is a technology that reduces the dimension of highly correlated multidimensional data and transforms them into a new variable system, principal components [28,29]. PCA was conducted using SIMCA© software (Sartorius Stedim Biotech., version 15, Umeå, Sweden). The Pearson correlation coefficient is a type of correlation analysis, a technique that identifies whether there is a correlation between two variables [30]. This can quantitatively evaluate the effect of one variable on another. Pearson correlation coefficients were obtained using Origin 2020 software (OriginLab, Northampton, MA, USA). IQAs and CQAs were used as variables. The Pearson correlation coefficient is the product of the covariance of two variables divided by the product of the standard deviation [31]. The Pearson correlation coefficient was calculated using Equation (7). The value of the Pearson correlation coefficient ranged from −1 to 1. An approximate value of −1 suggested a strong negative correlation, and an approximate value of 1 indicated a strong positive correlation. In addition, a value approximating 0 indicated that there was no correlation.

(7) $$r\;=\;\frac{\sum_{i}^{n}\left(X_{i}-\bar{X}\right)\left(Y_{i}-\bar{Y}\right)}{\sqrt{\sum_{i}^{n}{\left(X_{i}-\bar{X}\right)}^{2}}\sqrt{\sum_{i}^{n}{\left(Y_{i}-\bar{Y}\right)}^{2}}}$$

where *r* is the strength of the linear correlation between the *X* and *Y* variables, *n* is the number of samples, $\bar{X}$ is the average of *X* samples, and $\bar{Y}$ is the average of *Y* samples.

### 2.6. Initial Risk Assessment for the Manufacturing Process Development

The quality target product profile (QTPP) of the bilayer tablet was defined based on the XIGDUO™ XR (AstraZeneca Pharmaceuticals LP, Wilmington, DE, USA). As is shown in Table 1, QTPP includes the dosage form, dosage design, route of administration, dosage strength, pharmacokinetics, stability, drug product quality attributes, intermediate product quality attributes, and container closure system. A risk assessment was conducted to identify the high-risk process parameter that might have a significant effect on IQAs and CQAs. The initial risk assessment was evaluated by failure mode event analysis (FMEA), and the severity, probability, and detection were evaluated on a scale of 1–5 in terms of risk. The risk priority number (RPN) was calculated by multiplying the three severity, probability, and detectability, and RPN values of 1–19, 20–39, and 40–125 were classified as low, medium, and high risk, respectively.

## 3. Results and Discussion

### 3.1. Initial Risk Assessment for the Manufacturing Process Development

#### 3.1.1. Initial Risk Assessment for High-Shear Wet Granulation

The quality of the intermediate product after the unit process has an effect on the downstream process, which, in turn, affects the quality characteristics of the drug product [3,24]. The intrinsic dissolution rate may be significantly related to the drug product dissolution profile, which affects bioavailability. In addition, the intrinsic dissolution rate may have a greater correlation with the in vivo dissolution kinetics than the solubility test [22]. The size of the granules and the strength of the granules can affect fluidity, content uniformity, and solubility [32,33,34]. Granule fluidity parameters such as Carr’s index and the angle of repose are related to the assay, content uniformity, and dissolution. The density of the granules can affect compressibility [35]. Therefore, the quality characteristics of intermediate products such as the intrinsic dissolution rate, granule size, true density, bulk density, Carr’s index, the angle of repose, and granular strength should be evaluated throughout process development. The assay and content uniformity can affect the safety and efficacy of the drug product [36]. Undesired dissolution may result in unexpected bioavailability [36]. Inappropriate tablet hardness can affect safety and efficacy [37]. Friability of less than 1.0% *w*/*w* can reduce the impact on patient safety and efficacy [36]. Swelling property, weight gain, mass loss, and gel strength are related to dissolution, which affects bioavailability [38]. The wettability of tablets can be assessed by measuring the contact angle, and as the contact angle is directly related to dissolution, which affects bioavailability, it affects safety and efficacy [39]. Therefore, the quality characteristics of drug products such as assay, content uniformity, swelling property, weight gain, mass loss, gel strength, dissolution, hardness, friability, and contact angle should be evaluated throughout process development; herein, these characteristics were evaluated as response factors of DoE in the manufacturing process development.

The process parameters to be considered in the high-shear wet granulation process include the binding solvent spray rate, binding solvent amount, impeller speed, massing time, drying temperature, and drying time. Initial risk assessment was conducted to select process variables that have a significant influence on the quality characteristics of intermediate and drug products among process variables. Although the spray rate of the binder solvent may affect the quality characteristics of intermediate and drug products, it was classified as having medium and low risk, as it was used as a fixed value in the process. A high ratio of liquid binders increases residence time and torque, and forms more spherically shaped granules, owing to the formation of more liquid bridges [40]. In addition, the binder solvent affects the porosity and size of the granules [41,42]. Therefore, the binder solvent poses a high risk for intrinsic dissolution rate, granule size, granule strength, dissolution, true density, bulk density, Carr’s index, angle of repose, content uniformity, swelling property, weight gain, mass loss, gel strength, and contact angle. Inadequate impeller speed can lead to inadequate granule growth [11,12,13]. Inadequate granule growth leads to undesired granule porosity and strength [24]. In addition, the impeller speed creates a high shear between the particles, and the growth and densification of the granules proceeds [43,44]. For this reason, the impeller speed was classified as high risk because it can have a significant effect on the intrinsic dissolution rate, granule size, true density, bulk density, Carr’s index, angle of repose, granule strength, gel strength, dissolution, and contact angle. In addition, it was classified as medium risk because it can affect assay, content uniformity, swelling property, weight gain, mass loss, hardness, and friability. Failure to control the massing time can produce weak granules or granules of inadequate density [11,45,46]. In addition, as the massing time increases, high shear occurs between particles, thereby leadings to granule growth and densification. Therefore, the massing time was classified as a high risk because it can have a significant effect on the intrinsic dissolution rate, granule size, true density, bulk density, granule strength, and dissolution. Moreover, massing time was classified as medium risk because it can affect Carr’s index, angle of repose, assay, content uniformity, swelling property, weight gain, mass loss, gel strength, hardness, friability, and contact angle. Drying temperature and drying time can affect the quality characteristics of intermediate and drug products, but they are fixed based on previous experience and are therefore low risk. Based on the initial risk assessment, the impeller speed, massing time, and binder solvent were selected as CPPs.

#### 3.1.2. Initial Risk Assessment for Roller Compaction

As mentioned above, the intrinsic dissolution rate, granule size, ribbon density, bulk density, tap density, angle of repose, granule strength, and granule uniformity in roller compaction process were selected as the quality characteristics of the intermediate products. The assay, content uniformity, hardness, friability, dissolution, tablet C.U., and contact angle were selected as the quality characteristics of the drug product. IQAs and CQAs were evaluated as DoE response factors in the manufacturing process development.

The process parameters to be considered in roller compaction include feed screw speed, roller pressure, roller speed, roller gap, mill screen type, mill speed, mill screen size, and environment (temperature and RH). Roller pressure is important for processing parameters that determine powder cohesion [14]. Powder cohesion is directly related to ribbon density [14]. As the roller pressure increases, the force applied to the powder increases [16]. As the force increases, the air introduced into the powder is discharged, resulting in an increase in ribbon density and strength [16]. Strong ribbons produce stronger and larger granules after milling. This can affect granule flowability, content uniformity, and compressibility. Thus, roller pressure was classified as high risk because it can have a significant effect on the intrinsic dissolution rate, granule size, ribbon density, bulk density, tapped density, granule uniformity, tablet C.U., and dissolution. When the gap between the rolls increases, a thick ribbon is created. Even if the same force is applied to the ribbon, the degree of force transmission varies depending on the thickness of the ribbon; thus, the strength of the ribbon is lowered in the case of a thick ribbon, and smaller and weaker granules are produced [16]. Ultimately, the roller gap determines the ribbon density and affects the granule properties [47]. Therefore, the roller gap was classified as high risk for the intrinsic dissolution rate, granule size, ribbon density, bulk density, tap density, granule uniformity, tablet content uniformity, and dissolution. In addition, the contact angle was judged as medium risk. Roller speed is a variable that determines the throughput of the process. It can affect IQAs and CQAs but is low risk because it is fixed, based on previous experience. The mill screen type can affect the properties of the granules; however, generally, the mill screen type does not change significantly, so the effect on the CQAs and IQAs is low. The mill speed can affect the granule properties, but its risk is low. Mill screen size can affect the physical properties of granules, which can affect granule size and flowability [48]. Therefore, mill screen size was classified as high risk because it can have a significant effect on the intrinsic dissolution rate, granule size, granule strength, granule uniformity, assay, content uniformity, hardness, friability, dissolution, tablet C.U., and contact angle. The effect of the working environment on the CQAs and IQAs is low because the manufacturing facility maintains a constant temperature and humidity. Based on the initial risk assessment, roller pressure, roller gap, and mill screen size were selected as CPPs.

### 3.2. Effect of CPPs on the IQAs and CQAs of High-Shear Wet Granulation

#### 3.2.1. Effect of CPPs on the Intrinsic Dissolution Rate (*q*_1_)

The intrinsic dissolution rate was in the 5.63–7.32 mg·mm^−2^·min^−1^ range. The standard deviation was 0.44. An ANOVA was conducted to identify the individual influence of control factors (CPPs) on response factors (IQAs and CQAs). The individual influence of control factors is described by the reduced linear mathematical model, which is presented in Equation (8).

*q*_1_ = 0.1978*p*_1_ − 0.1576*p*_2_ + 0.0014*p*_3_ + 0.5776*p*_1_*p*_2_ − 0.1703*p*_1_^2^ − 0.1648*p*_2_^2^ + 0.3614*p*_3_^2^ (8)

A *p*-value less than 0.05 indicates that control factors had a significant effect on the response factors. The intrinsic dissolution rate ANOVA results showed that the *p*-values of all control factors were less than 0.05. The R^2^ value of the response factors (*q*_1_) was 0.9689, which indicates that the control factors had a significant effect on the response factors. According to Equation (8), massing time had a negative effect on the intrinsic dissolution rate. Larger-sized granules have a smaller water contact area than smaller-sized granules, so the disintegration rate is slow [24]. Therefore, larger granules have slow dissolution. In general, increasing the massing time could increase the granule size because it presents the mechanical energy required for mixing the powder [24]. Thus, the intrinsic dissolution rate could be negatively influenced by the massing time.

#### 3.2.2. Effect of CPPs on Granule Size (*q*_2_–*q*_6_)

The granule sizes (D_10_, D_50_, D_90_, D(3,2), and D(4,3)) were in the 17.00–37.77 µm, 29.30–286.07 µm, 181.07–1420.28 µm, 32.97–96.40 µm, and 121.67–690.33 µm ranges, respectively. The standard deviations were 7.79, 102.18, 412.17, 24.57, and 219.59, respectively. Based on the granule size results, we used an ANOVA to identify the individual influence of control factors (CPPs) on response factors (IQAs and CQAs). The individual influence of control factors is described by the reduced linear mathematical model, which is presented in Equations (9)–(13).

*q*_2_ = 0.7325*p*_1_ + 9.47*p*_3_ + 1.94*p*_1_^2^ + 5.01*p*_3_^2^ (9)

*q*_3_ = 10.48*p_1_* + 12.05*p*_2_ + 224.23*p*_3_ + 51.76*p*_1_*p_2_* + 44.25*p*_2_^2^ + 161.10*p*_3_^2^ (10)

*q*_4_ = 100.59*p*_2_ + 578.71*p*_3_ (11)

*q*_5_ = 2.31*p*_1_ + 2.53*p*_2_ + 30.05*p*_3_ + 3.40*p*_1_*p*_2_ + 4.16*p*_1_^2^ + 16.31*p*_3_^2^ (12)

*q*_6_ = 17.53*p*_1_ + 18.24*p*_2_ + 265.46*p*_3_ + 75.08*p*_1_*p*_2_ + 63.86*p*_2_^2^ + 124.95*p*_3_^2^ (13)

According to the ANOVA results regarding granule size, the *p*-values of all control factors were less than 0.05. The R^2^ values of the response factors (*q*_2_–*q*_6_) were 0.9706, 0.9895, 0.9338, 0.992, and 0.9787, respectively, which indicates that the control factors had a significant effect on the response factors. According to Equations (9)–(13), the binder solvent had a positive effect on granule size. This is because when a large amount of binding solvent is added to the powder bed, strong liquid bridges are formed between the particles, which increases the particle size [41,42].

#### 3.2.3. Effect of CPPs on True Density, Bulk Density, Carr’s Index, and Angle of Repose (*q*_7_–*q*_10_)

The true density, bulk density, Carr’s index, and angle of repose were in the 0.69–0.70g/mL, 0.052–0.059 g/mL, 19.75–26.59, and 27.4–43.3° ranges, respectively. The standard deviations of the true density and bulk density approximated 0.00. The standard deviations of Carr’s index and the angle of repose were 2.36 and 4.42, respectively. Based on the results of true density, bulk density, Carr’s index, and angle of repose results, an ANOVA was conducted to identify the individual influence of the control factors (CPPs) on the response factors (IQAs and CQAs). The individual influence of control factors is described by the reduced quadratic mathematical model and the reduced 2FI (factor of interaction) mathematical model, which is presented in Equations (14)–(17).

*q*_7_ = 0.0007*p*_1_ − 0.0005*p*_2_ + 0.0005*p*_3_ − 0.0027*p*_1_*p*_2_ + 0.0023*p*_2_*p*_3_ (14)

*q*_8_ = −0.0008*p*_1_ + 0.0001*p*_2_ − 0.0014*p*_3_ + 0.0025*p*_1_*p*_2_ − 0.0023*p*_3_^2^ (15)

*q*_9_ = 0.2000*p*_2_ + 4.03*p*_3_ + 0.6250*p*_2_*p*_3_ − 0.3923*p*_2_^2^ + 0.6577*p*_3_^2^ (16)

*q*_10_ = −0.6000*p*_1_ + 1.76*p*_2_ + 4.99*p*_3_ − 1.70*p*_1_*p*_2_ + 2.80*p*_1_*p*_3_ (17)

According to the ANOVA results regarding true density, bulk density, Carr’s index, and angle of repose, the *p*-values of all control factors were less than 0.05. The R^2^ values of the response factors (*q*_7_–*q*_10_) were 0.8815, 0.9171, 0.9937, and 0.9870, respectively, which indicates that the control factors had a significant effect on the response factors. According to Equation (14), the true density was positively affected by the impeller speed, binder solvent, and interaction between impeller speed and massing time. Bulk density was negatively affected by the impeller speed and binder solvent. If the impeller speed increases, the granule size may increase because granule coalescence and growth may occur [24,41,42,49]. In addition, when many binding solvents are added to the powder bed, strong liquid bridges are formed between the particles, which increases the particle size [41,42]. Larger granules have a lower bulk density than smaller granules because less can fit in the same volume. Therefore, impeller speed and binder solvent can negatively affect bulk density. According to Equation (16), an increase in the binder solvent increases Carr’s index, which results in a decrease in the granule flowability. According to Equation (17), an increase in the binder solvent increases the angle of repose and decreases granule flowability.

#### 3.2.4. Effect of CPPs on Granule Strength (*q*_11_)

The granule strength was in the 0.05–0.69 N∙sec range. The standard deviation was 0.17. Based on the results of the granule strength analysis, an ANOVA was conducted to identify the individual influence of control factors (CPPs) on response factors (IQAs and CQAs). The individual influence of control factors is described by the reduced 2FI (factor of interaction) mathematical model, which is presented in Equation (18).

*q*_11_ = 0.0900*p*_1_ − 0.0400*p*_2_ − 0.0725*p*_3_ − 0.1400*p*_1_*p*_2_ − 0.2200*p*_1_*p*_3_ (18)

According to the ANOVA results regarding granule strength, the *p*-values of all control factors were less than 0.05. The R^2^ value of the response factors (*q*_11_) was 0.9752, which indicates that the control factors had a significant effect on the response factors. According to Equation (18), the impeller speed increases granule strength. This might increase the impeller speed, which increases granule hardness because increasing the impeller speed applies high shearing to granules, thereby leading to increased granule density and strength [43,44].

#### 3.2.5. Effect of CPPs on the Swelling Property (*q*_12_, *q*_13_, and *q*_14_)

The swelling properties (1 h, 3 h, and 5 h) were in the 29.01–32.34%, 46.49–51.08%, and 62.11–64.42% ranges, respectively. The standard deviations were 0.99, 1.38, and 0.70, respectively. Based on the swelling property results, an ANOVA was conducted to identify the individual influence of control factors (CPPs) on response factors (IQAs and CQAs). The individual influence of control factors is described by the reduced 2FI (factor of interaction) mathematical model, which is presented in Equations (19)–(21).

*q*_12_ = −0.3152*p*_1_ + 1.07*p*_2_ + 0.6223*p*_3_ (19)

*q*_13_ = −0.4956*p*_1_ − 0.7939*p*_2_ − 0.8415*p*_3_ + 0.4619*p*_1_*p*_2_ − 1.22*p*_1_*p*_3_ − 1.20*p*_2_*p*_3_ (20)

*q*_14_ = 0.7322*p*_1_ + 0.1269*p*_2_ − 0.1833*p*_3_ − 0.1572*p*_1_*p*_2_ − 0.1929*p*_1_*p*_3_ + 0.6752*p*_2_*p*_3_ − 0.0763*p*_1_^2^ − 0.0763*p*_2_^2^ + 0.1197*p*_3_^2^ (21)

According to the ANOVA results regarding the swelling property, the *p*-values of all control factors were less than 0.05. The R^2^ values of the response factors (*q*_12_, *q*_13_, and *q*_14_) were 0.9804, 0.9532, and 0.9998, respectively, which indicates that the control factors had a significant effect on the response factors. According to Equation (19), an increase in massing time increases the swelling property. In addition, as the impeller speed increased, the swelling property increased at 5 h. According to Equation (3), increased erosion increases the swelling property. The erosion rate increases as the particle size increases [50,51]. If the massing time and impeller speed are increased, the granule size may increase because granule coalescence and growth may occur [24,41,42,49]. Therefore, the impeller speed and massing time increased the swelling property because the erosion rate increased as the impeller speed and massing time increased.

#### 3.2.6. Effect of CPPs on Weight Gain (q_15_, q_16_, and q_17_) and Mass Loss (*q*_18_, *q*_19_, and *q*_20_)

The weight gains (1 h, 3 h, and 5 h) were in the 54.90–69.57%, 55.13–75.59%, and 44.55–75.68% ranges, respectively. The standard deviations of weight gain were 3.88, 5.78, and 7.85, respectively. Mass losses (1 h, 3 h, and 5 h) were in the 24.10–33.76%, 45.08–50.71%, and 68.29–77.50% ranges, respectively. The standard deviations of mass loss were 2.51, 1.64, and 2.48, respectively. Based on the weight gain and mass loss results, an ANOVA was conducted to identify the individual influence of control factors (CPPs) on response factors (IQAs and CQAs). The individual influence of control factors is described by the reduced 2FI (factor of interaction) mathematical model, which is presented in Equations (22)–(27).

*q*_15_ = 1.35*p*_1_ − 4.43*p*_2_ + 0.5916*p*_3_ + 2.92*p*_1_*p*_2_ (22)

*q*_16_ = 2.06*p_1_* − 5.23*p*_2_ + 0.1889*p*_3_ + 5.12*p*_1_*p*_2_ − 4.58*p_1_p_3_* + 2.41*p*_2_*p*_3_ (23)

*q*_17_*=* −0.9555*p*_1_ − 8.39*p*_2_ − 0.7901*p*_3_ + 3.97*p*_1_*p*_2_ + 7.25*p*_2_*p*_3_ (24)

*q*_18_ = 0.5310*p*_1_ + 2.88*p*_2_ − 0.3433*p*_3_ + 1.15*p*_1_*p*_2_ − 1.*71p*_2_*p*_3_ (25)

*q*_19_ = −0.3141*p*_1_ + 1.80*p*_2_ − 0.7558*p*_3_ − 0.7325*p*_1_*p*_2_ − 1.01*p*_2_*p*_3_ (26)

*q*_20_ = −0.1045*p*_1_ + 2.66*p*_2_ − 1.58*p*_3_ − 0.9154*p*_1_*p*_2_ − 1.07*p*_2_*p*_3_ (27)

According to the ANOVA results for weight gain and mass loss, the *p*-values of all control factors were less than 0.05. The R^2^ values of the response factors (*q*_15_, *q*_16_, and *q*_17_) were 0.9892, 0.9938, and 0.9838, respectively, which indicates that the control factors had a significant effect on the response factors. The R^2^ values of the response factors (q_18_, q_19_, and q_20_) were 0.9841, 0.9911, and 0.9842, respectively, which indicates that the control factors had a significant effect on the response factors. According to Equations (22)–(24), massing time decreases weight gain. Generally, an increase in massing time leads to a decrease in granule porosity [52,53,54]. When the granule porosity decreases, water penetration into the tablet decreases, which negatively affects weight gain. In addition, when hydrophilic polymers are used, the weight gain increases rapidly due to the rapid absorption of water at the beginning; however, as time passes, the gel layer acts as a barrier, so the weight gain is slightly changed by absorbing the aqueous medium slowly.

According to Equations (25)–(27), massing time increases mass loss. According to the Equation (5), increased tablet erosion increases mass loss. The erosion rate increases as the particle size increases [50,51]. If the massing time is increased, the granule size may increase because granule coalescence and growth may occur [24,41,42,49]. Therefore, massing time increases mass loss because the erosion rate increases as the massing time increases.

#### 3.2.7. Effect of CPPs on Gel Strength (*q*_21_, *q*_22_, and *q*_23_)

The gel strengths (1 h, 3 h, and 5 h) were in the 1.41–13.29 N∙sec, 1.97–10.15 N∙sec, and 1.58–7.22 N∙sec, ranges respectively. The standard deviations were 3.53, 2.48, and 1.85, respectively. Based on the results of gel strength analysis, an ANOVA was conducted to identify the individual influence of control factors (CPPs) on response factors (IQAs and CQAs). The individual influence of control factors is described by the reduced 2FI (factor of interaction) mathematical model, which is presented in Equations (28)–(30).

*q*_21_ = +1.17*p*_1_ − 1.56*p*_2_ + 4.05*p*_3_ (28)

*q*_22_ = −0.1167*p*_1_ − 1.49*p*_2_ + 0.5977*p*_3_ − 0.6932*p*_1_*p*_2_ − 2.78*p*_1_*p*_3_ − 2.74*p*_2_*p*_3_ (29)

*q*_23_ = −0.1729*p*_1_ − 0.7796*p*_2_ + 0.2926*p*_3_ + 2.08*p*_1_*p*_2_ − 2.05*p*_1_*p*_3_ + 1.18*p*_2_*p*_3_ (30)

According to the ANOVA results regarding gel strength, the *p*-values of all control factors were less than 0.05. The R^2^ values of the response factors (*q*_21_, *q*_22_, and *q*_23_) were 0.9287, 0.9696, and 0.9501, respectively, which indicates that the control factors had a significant effect on the response factors. According to Equations (28)–(30), the gel strength at 1 h was positively affected by the impeller speed and binder solvent, while at 3 h it was negatively affected by the interaction between the impeller speed and binder solvent and the interaction between the massing time and the binder solvent. According to Equation (30), the gel strength at 5 h was positively affected by the interaction between the impeller speed and massing time.

#### 3.2.8. Effect of CPPs on Dissolution (*q*_24_, *q*_25_, and *q*_26_)

The dissolution profiles (1 h, 3 h, and 10 h) were in the 41.66–46.42%, 74.55–82.57%, and 104.06–108.97% ranges, respectively. The standard deviations were 1.50, 2.15, and 1.50, respectively. Based on the dissolution results, an ANOVA was conducted to identify the individual influence of control factors (CPPs) on response factors (IQAs and CQAs). The individual influence of control factors is described by the reduced 2FI (factor of interaction) mathematical model, which is presented in Equations (31)–(33).

*q*_24_ = −0.2500*p*_1_ + 0.4275*p*_2_ + 0.6700*p*_3_ − 1.03*p*_1_*p*_2_ − 1.87*p*_1_*p*_3_ − 1.20*p*_2_*p*_3_ (31)

*q*_25_ = −0.9363*p*_1_ + 0.1425*p*_2_ + 0.7788*p*_3_ − 1.40*p*_1_*p*_3_ − 3.18*p*_2_*p*_3_ (32)

*q*_26_ = 0.1137*p*_1_ + 0.4375*p*_2_ + 0.09386*p*_3_ + 1.85*p*_1_*p*_2_ + 0.6100*p*_1_*p*_3_ − 1.87*p*_2_*p*_3_ (33)

According to the ANOVA results regarding dissolution, the *p*-values of all control factors were less than 0.05. The R^2^ values of the response factors (*q*_24_, *q*_25_, and *q*_26_) were 0.9400, 0.9338, and 0.9833, respectively, which indicates that the control factors had a significant effect on the response factors. At the initial 1 h, the dissolution was negatively influenced by the interaction between the impeller speed and the binder solvent. If the impeller speed increases, the granule size may increase because granule coalescence and growth may occur [24,41,42,49]. Furthermore, when a large amount of binding solvent is added to the powder bed, strong liquid bridges are formed between the particles, which increases the particle size [41,42]. The increased granule size has a smaller surface area compared to the smaller granule size. Larger-sized granules have a smaller water contact area than the smaller-sized granules, so the disintegration rate is slow [24]. Therefore, larger granules have slow dissolution. Thus, the dissolution could be negatively influenced by the massing time and binder solvent. At 3 h and 10 h, the interaction between massing time and binder solvent decreased the dissolution. Increasing the massing time could increase the granule size because it presents the mechanical energy required for mixing the powder [24]. Furthermore, when a large amount of binding solvent is added to the powder bed, strong liquid bridges are formed between the particles, which increases the particle size [41,42]. Large granules have a smaller surface area than small granules, so drug release is slow [24]. Thus, the dissolution could be negatively influenced by the massing time and amount of binder solvent.

#### 3.2.9. Effect of CPPs on the Contact Angle (*q*_27_)

The contact angle was in the 3.88–10.76 θ/sec range. The standard deviation was 1.80. Based on the results of the contact angle, an ANOVA was conducted to identify the individual influence of control factors (CPPs) on response factors (IQAs and CQAs). The individual influence of control factors is described by the reduced 2FI (factor of interaction) mathematical model, which is presented in equation (34).

*q*_27_ = −0.7754*p*_1_ − 0.6704*p*_2_ + 0.3488*p*_3_ + 2.13*p*_1_*p*_2_ + 2.00*p*_2_*p*_3_ (34)

According to the ANOVA results regarding the contact angle, the *p*-values of all control factors were less than 0.05. The R^2^ value of the response factors (*q*_27_) was 0.9580 which indicates that the control factors had a significant effect on the response factors. According to Equation (34), the impeller speed and massing time decrease the contact angle. If the massing time and impeller speed increase, the granule size may increase because granule coalescence and growth may occur [24,41,42,49]. The increased granule size results in a smaller surface area than smaller granule size. Therefore, larger granules have a smaller water contact area than smaller sized granules, so the wetting rate is slow, which negatively affects the contact angle.

### 3.3. Effect of CPPs on the IQAs and CQAs of Roller Compaction

#### 3.3.1. Effect of CPPs on the Intrinsic Dissolution Rate (*d*_1_)

The intrinsic dissolution rate was in the 0.0138–0.0198 μg·mm^−2^·min^−1^ range. The standard deviation was approximately 0.00. Based on the results of the intrinsic dissolution rate, an ANOVA was conducted to identify the individual influence of control factors (CPPs) on response factors (IQAs and CQAs). The individual influence of control factors is described by the reduced linear mathematical model, which is presented in Equation (35).

*d*_1_ = −0.009*c*_1_ − 0.0023*c*_3_ (35)

According to the ANOVA results regarding the intrinsic dissolution rate, the *p*-values of all control factors were less than 0.05. The R^2^ values of the response factors (*d*_1_) were 0.9225, which indicates that the control factors had a significant effect on the response factors. According to Equation (35), the roller pressure and mill screen size decrease the intrinsic dissolution rate. It is possible that increasing the roller pressure and increasing the mill screen size generated large granules [48,55]. Large granules have a smaller surface area than small granules, so drug release is slow [24]. Therefore, the roller pressure and mill screen size can decrease the intrinsic dissolution rate.

#### 3.3.2. Effect of CPPs on Granule Size (*d*_2_–*d*_6_)

The granule sizes (D_10_, D_50_, D_90_, D(3,2), and D(4,3)) were in the 8.62–10.10 µm, 59.80–68.09 µm, 245.78–317.14 µm, 14.71–15.70 µm, and 106.10–128.54 µm ranges, respectively. The standard deviations were 0.51, 2.19, 19.96, 5.77, and 19.96, respectively. Based on the granule size results, an ANOVA was conducted to identify the individual influence of control factors (CPPs) on response factors (IQAs and CQAs). The individual influence of control factors is described by the reduced 2FI (factor of interaction) mathematical model, which is presented in Equations (36)–(40).

*d*_2_ = 0.4225*c*_1_ − 0.0075*c*_2_ + 0.0900*c*_3_ + 0.7075*c*_2_*c*_3_ (36)

*d*_3_ = 2.12*c*_1_ − 1.23*c*_2_ + 1.33*c*_3_ (37)

*d*_4_ = 23.14*c*_1_ + 8.92*c*_3_ (38)

*d*_5_ = 0.3125*c*_1_ + 0.0687*c*_2_ + 0.1462*c*_3_ (39)

*d*_6_ = 5.46*c*_1_ + 4.88*c*_3_ (40)

According to the ANOVA results regarding granule size, the *p*-values of all control factors were less than 0.05. The R^2^ values of the response factors (*d*_2_–*d*_6_) were 0.9553, 0.9178, 0.8821, 0.9519, and 0.9190 indicating that the control factors had a significant effect on the response factors. According to equation (36), the interaction between the roller gap and mill screen size increases the granule size. According to equations (37)–(40), the roller pressure increases the granule size. Generally, increasing the roller pressure and increasing the mill screen size generate large granules [48,55]. Therefore, the roller pressure and mill screen size increase the granule size.

#### 3.3.3. Effect of CPPs on Ribbon Density, Bulk Density, and Tapped Density (*d*_7_, *d*_8_, and *d*_9_)

The ribbon density, bulk density, and tapped density were in the 1.01–1.26 g/cm^3^, 0.046–0.055 g/mL, and 0.064–0.067 g/mL ranges, respectively. The standard deviation of the ribbon density was 0.08. The standard deviations of the bulk density and tapped density were approximate 0.00. Based on the ribbon density, bulk density, and tapped density results, an ANOVA was conducted to identify the individual influence of control factors (CPPs) on response factors (IQAs and CQAs). The individual influence of control factors is described by the reduced linear mathematical model and generates the reduced quadratic mathematical model, which is presented in Equations (41)–(43).

*d*_7_ = 0.1037*c*_1_ − 0.0163*c*_2_ + 0.0100*c*_3_ (41)

*d*_8_ = −0.0016*c*_1_ − 0.0008*c*_2_ − 0.0021*c*_3_ + 0.0027*c*_3_^2^ (42)

*d*_9_ = 0.0001*c*_1_ − 0.0003*c*_2_ − 0.0001*c*_3_ − 0.0002*c*_1_*c*_2_ − 0.0008*c*_2_*c*_3_ + 0.0008*c*_1_^2^ + 0.0005*c*_2_^2^ + 0.0012*c*_3_^2^ (43)

According to the ANOVA results regarding ribbon density, bulk density, and tapped density, the *p*-values of all control factors were less than 0.05. The R^2^ values of the response factors (*d*_7_, *d*_8_, and *d*_9_) were 0.9816, 0.9575, and 0.9779, respectively, indicating that the control factors had a significant effect on the response factors. According to Equation (41), roller pressure increases ribbon density. Generally, an increase in roller pressure increases ribbon density [15]. Therefore, ribbon density is positively influenced by roller pressure. According to Equation (42), the mill screen size decreases the bulk density. Generally, large pore size sieves generate large-sized granules [55]. Larger granules have a lower blk density than smaller granules because less can fit in the same volume. Thus, the mill screen size decreases the bulk density. According to Equation (43), an increase in the roller gap decreases the tapped density.

#### 3.3.4. Effect of CPPs on Granule Strength and Granule Uniformity (*d*_10_ and *d*_11_)

The granule strength and granule uniformity were in the 0.04–0.34 N∙sec and 1.22–3.90% RSD ranges, respectively. The standard deviations were 0.09 and 0.7, respectively. Based on the granule strength and granule uniformity results, an ANOVA was conducted to identify the individual influence of control factors (CPPs) on response factors (IQAs and CQAs). The individual influence of control factors is described by the reduced 2FI (factor of interaction) mathematical model, which is presented in Equations (44)–(45).

*d*_10_ = 0.1094*c*_1_ + 0.0406*c*_2_ (44)

*d*_11_ = 0.1575*c*_1_ − 0.0350*c*_2_ + 0.3025*c*_3_ + 1.17*c*_2_*c*_3_ (45)

According to the ANOVA results regarding granule strength and granule uniformity, the *p*-values of all control factors were less than 0.05. The R^2^ values of the response factors (*d*_10_ and *d*_11_) were 0.9687 and 0.9435, respectively, which indicates that the control factors had a significant effect on the response factors. According to Equation (44), roller pressure increases granule strength. Generally, increasing roller pressure results in increased ribbon density and thus granules harder [15]. Therefore, the granule strength was positively influenced by the roller pressure. According to Equation (45), granule uniformity was positively affected by the mill screen size and the interaction between the roller gap and mill screen size.

#### 3.3.5. Effect of CPPs on Tablet C.U. (*d*_12_)

The tablet C.U. was in the 2.08–4.10% RSD range. The standard deviation was 0.53. Based on the tablet C.U. result, an ANOVA was conducted to identify the individual influence of control factors (CPPs) on response factors (IQAs and CQAs). The individual influence of control factors is described by the linear mathematical model, which is presented in Equation (46).

*d*_12_ = −0.5375*c*_1_ + 0.2537*c*_2_ − 0.3562*c*_3_ (46)

According to the ANOVA results regarding tablet C.U., the *p*-values of all control factors were less than 0.05. The R^2^ value of the response factors (*d*_12_) was 0.9641, which indicates that the control factors had a significant effect on the response factors. According to Equation (46), the tablet C.U. was negatively affected by roller pressure and mill screen size. Increasing the roller pressure and the mill screen size generated large granules [48,55]. In general, the smaller the particle size, the better the content uniformity [56]. Thus, roller pressure and mill screen size had a negative effect on tablet C.U.

#### 3.3.6. Effect of CPPs on Dissolution (*d*_13_–*d*_15_)

The dissolution profiles (5 min, 10 min, and 15 min) were in the 37.19–65.78%, 53.56–83.15%, and 77.92–93.87% ranges, respectively. The standard deviations were 10.77, 10.19, and 5.66, respectively. Based on the dissolution results, an ANOVA was conducted to identify the individual influence of control factors (CPPs) on response factors (IQAs and CQAs). The individual influence of control factors is described by the reduced linear mathematical model, which is presented in Equations (47)–(49).

*d*_13_ = −3.93*c*_1_ − 12.98*c*_3_ (47)

*d*_14_ = −4.17*c*_1_ − 12.43*c*_3_ (48)

*d*_15_ = −2.15*c*_1_ − 6.83*c*_3_ (49)

According to the ANOVA results regarding dissolution, the *p*-values of all control factors were less than 0.05. The R^2^ values of the response factors (*d*_13_–*d*_15_) were 0.9057, 0.9459, and 0.9144, respectively, which indicates that the control factors had a significant effect on the response factors. According to Equations (47)–(49), dissolution was negatively affected by mill screen size. Generally, large pore size sieves generate large-sized granules. Large granules have a smaller surface area than small granules, so drug release is slow [24]. Therefore, dissolution was negatively affected by the mill screen size.

#### 3.3.7. Effect of CPPs on the Contact Angle (*d*_16_)

The contact angle was in 6.07–7.89 θ/s range. The standard deviation was 0.41. Based on the contact angle result, an ANOVA was conducted to identify the individual influence of control factors (CPPs) on response factors (IQAs and CQAs). The individual influence of control factors is described by the linear mathematical model, which is presented in Equation (50).

*d*_16_ = −0.2525*c*_1_ − 0.4337*c*_3_ (50)

According to the ANOVA results regarding the contact angle, the *p*-values of all control factors were less than 0.05. The R^2^ value of the response factors (*d*_16_) was 0.8746, which indicates that the control factors had a significant effect on the response factors. According to Equation (50), the mill screen size decreases the contact angle. The increased granule size results in a smaller surface area compared to the smaller granule size. Therefore, the larger granules have a smaller water contact area than the smaller sized granules, so the wetting rate is slow, which negatively affects the contact angle. Generally, large pore size sieves generate large-sized granules [55]. Therefore, mill screen size had a negative effect on the contact angle.

### 3.4. Design Spaces of Process Parameters

#### 3.4.1. Design Space of High-Shear Wet Granulation

**Figure 1:**
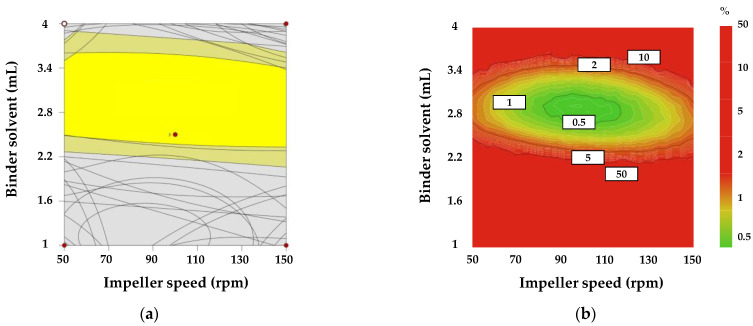
(**a**) Design space for high-shear wet granulation by combining response factors based on optimization conditions: the massing time was fixed at 30 s. The yellow area represents the 95% confidence interval. (**b**) Results of the Monte Carlo simulation for high-shear wet granulation: the massing time was fixed at 30 s. The intensity bar stands for the probability of failure. The green area represents a quality certainty of 99.9%.

The optimization conditions of response factors for high-shear wet granulation process were as follows: optimal range of angle of repose (30.5–40.5°); optimal range of a granule size D_10_ (18–36.8 μm); optimal range of D_50_ (30–280 μm); optimal range of a granule size D_90_ (400–1200 μm); optimal range of D(3,2) (33.2–95.4 μm); optimal range of a granule size D(4,3) (170–680 μm); optimal range of granule strength (0.15–0.65 N∙sec); optimal ranges of gel strength at 1 h (3.78–11.25 N∙sec), 3 h (2.61–8.61 N∙sec), and 5 h (1.59–7.21 N∙sec); optimal range of weight gain at 5 h (45.07–75.07%); optimal range of the contact angle (4.95–9.95 θ/s). The intrinsic dissolution rate, true density, bulk density, Carr’s index, dissolution at 1 h, 3 h, and 10 h, the swelling property at 1 h, 3 h, and 5 h, the weight gain at 1 h and 3 h, and mass loss at 1 h, 3 h, and 5 h results satisfied both the upper and lower limits of the target value and were excluded from the design space. The response factors were combined to produce a design space with a 95% confidence interval. Figure 1a shows the design space for high-shear wet granulation. The yellow area represents the 95% confidence interval. To evaluate the robustness of the design space and the risk of uncertainty in model predictions, a Monte Carlo simulation was conducted using the MODDE^®^ software (Sartorius Stedim Biotech., version 12.0.1, Umeå, Sweden). Figure 1b shows the result of the Monte Carlo simulation with the probability of failure. The green area represents a quality certainty of 99.9%, which indicates a 0.1% probability of failure. In contrast, the red area represents a higher probability of failure. As a result, the roller pressure had 99.9% quality certainty in an impeller speed range of approximately 50–150 rpm and a binder solvent range of approximately 2.3–3.4 mL when the massing time was fixed at 30 s.

#### 3.4.2. Design Space of Roller Compaction

**Figure 2:**
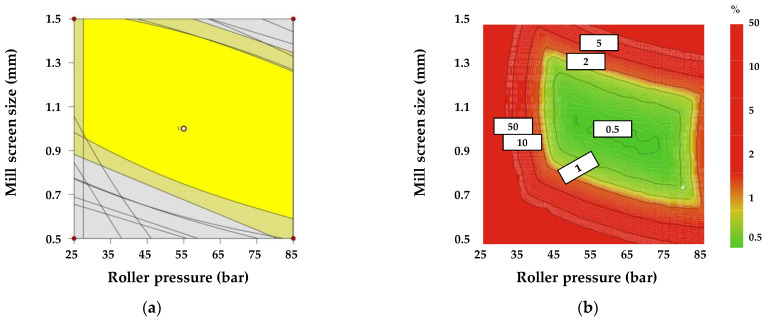
(**a**) Design space for roller compaction by combining response factors based on optimization conditions: the roller gap was fixed at 1.8 mm. The yellow area represents the 95% confidence interval. (**b**) Results of the Monte Carlo simulation for roller compaction: the roller gap was fixed at 1.8 mm. The intensity bar stands for the probability of failure. The green area represents a quality certainty of 99.9%.

The optimization conditions of the response factors for the roller compaction process were as follows: optimal range of intrinsic dissolution rate (0.0014–0.01847 μg/min/mm^2^); optimal range of D_50_ (61.04–68.08 μm); optimal range of a granule size D_90_ (400–1200 μm); optimal range of D(3,2) (33.2.95.4 μm); optimal range of granule strength (0.0096–0.296 N∙sec); optimal range of dissolution at 5 min (37.5–64.28%); optimal range of dissolution at 10 min (57.43–82.10%). The ribbon density, bulk density, tapped density, granule size D_10_, granule size(3,2), granule size(4,3), granule uniformity, tablet C.U., dissolution at 15, 30, 45, and 60 min, and contact angle results satisfied both the upper and lower limits of the target value and were excluded from the design space. The response factors were combined to produce a design space with a 95% confidence interval. Figure 2a shows the design space for roller compaction. The yellow area represents the 95% confidence interval. To evaluate the robustness of the design space and the risk of uncertainty in model predictions, a Monte Carlo simulation was conducted using the MODDE^®^ software (Sartorius Stedim Biotech., version 12.0.1, Umeå, Sweden). Figure 2b shows the results of the Monte Carlo simulation with the probability of failure. The green area represents a quality certainty of 99.9%, which suggests a 0.1% probability of failure. In contrast, the red area represents a higher probability of failure. As a result, roller pressure had 99.9% quality certainty in an approximate 45–85 bar range and the mill screen size in an approximate 0.7–1.3 mm range when the roller gap was fixed at 1.8 mm.

### 3.5. Multivariate Analysis between IQAs and CQAs

As the pharmaceutical process consists of unit operations, the intermediate product after the unit process affects the downstream process. Therefore, if we confirm the relationship between IQAs and CQAs, the latter can be predicted through the former. As DoE can process only a limited number of variables, the correlation between IQAs and CQAs was confirmed using MVA that can analyze multiple variables simultaneously. In this study, we used the Pearson correlation coefficient and PCA.

#### 3.5.1. Correlation between IQAs and CQAs of High Shear Wet Granulation Process

**Figure 3:**
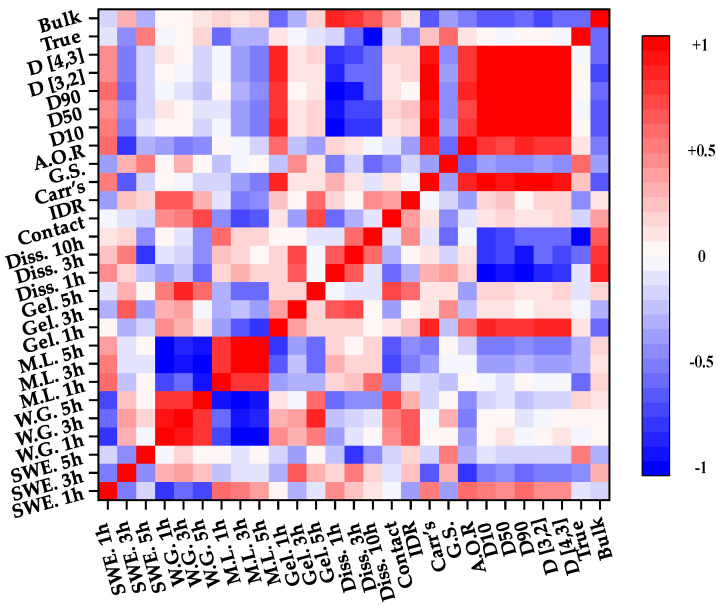
Pearson correlation coefficient of the high-shear wet granulation process. Bulk: bulk density; True: true density; A.O.R.: angle of repose; G.S.: granule strength; Carr’s: Carr’s index; IDR: intrinsic dissolution rate; Contact: contact angle; Diss.: dissolution; Gel.: gel strength; M.L.: mass loss; W.G.: weight gain; SWE.: swelling property.

Pearson correlation coefficient analysis was performed to evaluate the correlation among the response factors. The correlation coefficient value ranged from −1 to 1. Each value indicated the following correlations: −1, negative correlation; 0, uncorrelation; 1, positive correlation. Figure 3 visualizes the correlation among the response factors as a heat map. The red color indicates a value of 1, and the blue color indicates a value of −1.

Figure 3 shows the correlation between IQAs and CQAs in the high-shear wet granulation process. According to Figure 3, we confirmed that dissolution had a positive correlation with the intrinsic dissolution rate, Carr’s index, bulk density, and angle of repose. On the other hand, dissolution had a negative correlation with granule strength, granule size, and true density. In particular, the relationship between D_10_ and dissolution at 1 h and the relationship between bulk density and dissolution at 1 h were strongly correlated. This means that as the granule size decreases, dissolution and bulk density increase. In addition, as is shown in Figure 3, the bulk density and granule size have a negative correlation.

To evaluate the relationship between IQAs and CQAs, PCA was conducted using the SIMCA© software (Sartorius Stedim Biotech., version 15, Umeå, Sweden). The first, second, third, and fourth PCs explained 32.3%, 26.0%, 14.5%, and 12.2% of the overall variability, respectively, and the sum of these PCs accounted for 85% of the total. Points gathered together indicated correlation, and points located opposite to each other indicated that they had negative relationships.

**Figure 4:**
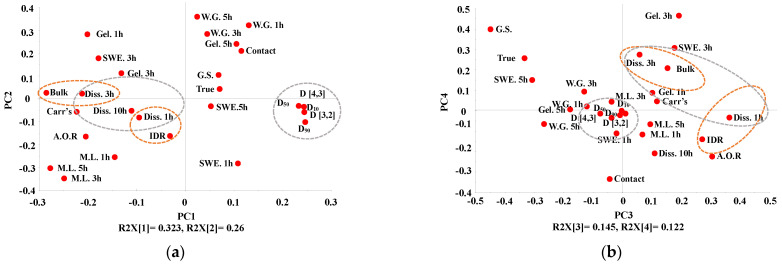
Score scatter plot of high-shear wet granulation: (**a**) PC1 and PC2, (**b**) PC3 and PC4. The groups within the gray dotted lines located opposite to each other, indicate a negative correlation. The groups within the orange dotted lines indicate positive correlations with each other. Bulk: bulk density; True: true density: A.O.R: angle of repose; G.S.: granule strength; Carr’s: Carr’s index; IDR: intrinsic dissolution rate; Contact: contact angle; Diss.: dissolution; Gel.: gel strength; M.L.: mass loss; W.G.: weight gain; SWE.: swelling property.

Figure 4a shows the loading plot with PC1 and PC2 of high-shear wet granulation. As is shown in Figure 4a, the granule size, contact angle, weight gain, granule strength, gel strength at 5 h, and swelling property at 5 h have positive loading values in PC1. In contrast, the intrinsic dissolution rate, gel strength at 1 h and 3 h, swelling property at 1 h and 3 h, bulk density, dissolution, angle of repose, Carr’s index, and mass loss have negative loading values in PC1. In addition, dissolution and granule size, which are located opposite to each other, have a negative correlation. In the case of PC2, granule size, bulk density, swelling property at 5 h, and true density near the zero line indicate that they have a smaller effect on PC2. Dissolution, Carr’s index, angle of repose, mass loss, swelling property at 1 h, and granule size have negative values in PC2. In addition, the intrinsic dissolution rate, weight gain, contact angle, granule strength, swelling property at 3 h, gel strength at 1 h and 5 h, and true density had positive values in PC2.

Figure 4b shows the loading plot with PC3 and PC4 of high-shear wet granulation. According to Figure 4b, the mass loss, dissolution, intrinsic dissolution rate, angle of repose, swelling property at 3 h, gel strength at 3 h, and bulk density had positive values in PC3. In contrast, the granule strength, true density, and swelling property at 5 h were negative for PC3. In addition, granule size and weight gain, which are located near the zero line, indicate that they had a smaller effect on PC3. Moreover, the granule size and weight gain are located near the zero line in PC4, indicating that they had a smaller effect on PC4. The contact angle, bulk density, angle of repose, and intrinsic dissolution rate had negative values in PC4. On the other hand, the granule strength, true density, gel strength at 3 h, and swelling property at 3 h were positive for PC4.

**Figure 5:**
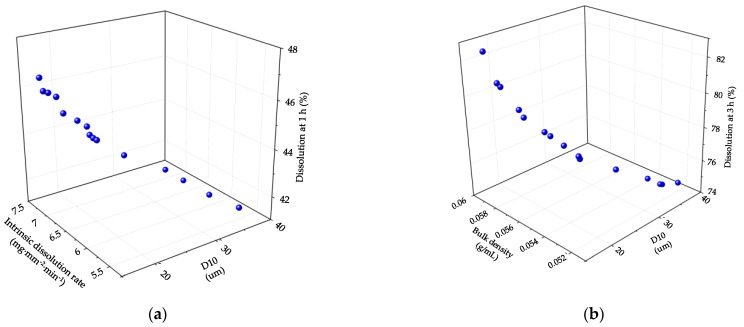
3D plot of high-shear wet granulation process: (**a**) relationship among D_10_, intrinsic dissolution rate, and dissolution at 1 h, (**b**) relationship among D_10_, bulk density, and dissolution at 3 h.

As dissolution may be significantly related to the bioavailability of drug products, it is important to satisfy the desired target of dissolution in the development of formulation and manufacturing processes [36]. Therefore, dissolution in CQAs was used to establish the control strategy for the high-shear wet granulation process. Based on the Pearson correlation coefficient and PCA results, we confirmed that in the high-shear wet granulation process, there was a strong relationship among granule size, intrinsic dissolution rate, and dissolution at 1 h, as well as a strong relationship among granule size, bulk density, and dissolution at 3 h. Figure 5 shows 3D plots representing the relationships among D_10_, intrinsic dissolution rate, and dissolution at 1 h, and the relationships among D_10_, bulk density, and dissolution at 3 h.

As is shown in Figure 5a, the dissolution profile at 1 h had a positive correlation with the intrinsic dissolution rate and a negative correlation with granule size. The intrinsic dissolution rate is significantly correlated with the drug product dissolution profile, which affects bioavailability [22]. The intrinsic dissolution rate refers to the rate of drug release from a constant surface, and a fast intrinsic dissolution rate indicates that the dissolution is faster. Therefore, the intrinsic dissolution rate has a positive correlation with dissolution. Granule size can affect solubility [32,33,34]. This is because the surface area varies depending on the granule size. The smaller the granule size, the larger the surface area, which in turn leads to a faster drug release owing to the larger area available for the granules and water to come into contact. Therefore, the smaller the granule size, the faster the drug is released [24], so there is a negative correlation between the granule size and the dissolution profile. As is shown in Figure 5b, the dissolution profile at 3 h had a positive correlation with bulk density and a negative correlation with granule size. This is because small granules have a larger surface area than large granules, thus drug release is fast [24]. Bulk density refers to the amount of granules that fit in the same volume. Therefore, smaller-sized granules have a larger bulk density because more can fit in the same volume. Thus, granule size has a negative correlation with bulk density. This means that monitoring IQAs in the high-shear wet granulation process can present target CQAs in real-time, except for the drug product experiments after the process.

#### 3.5.2. Correlation between the IQAs and CQAs of the Roller Compaction Process

**Figure 6:**
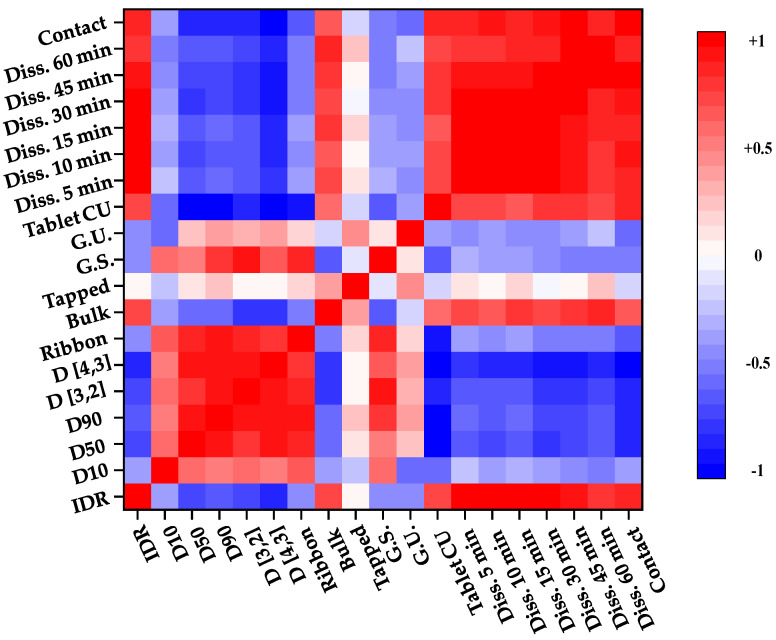
Pearson correlation coefficient of roller compaction process. Contact: contact angle; Diss.: dissolution; G.U.: granule uniformity; G.S.: granule strength; Tapped: tapped density; Bulk: bulk density; Ribbon: ribbon density; IDR: intrinsic dissolution rate.

Figure 6 shows the correlation between IQAs and CQAs in the roller compaction process. According to Figure 6, dissolution had a strong positive correlation with the intrinsic dissolution rate and bulk density and a strong negative correlation with granule size and ribbon density. In particular, there was strong negative correlation between granule size and dissolution. This means that as the granule size decreases, dissolution increases. Additionally, tapped density was less correlated with dissolution, as the value was approximately 0. The contact angle had a strong positive correlation with the intrinsic dissolution rate and bulk density and a strong negative correlation with granule size and ribbon density.

The first, second, and third PCs explained 65.1%, 13.5%, and 10.3% of the overall variability, respectively, and the sum of these PCs accounted for 88.9% of the total.

**Figure 7:**
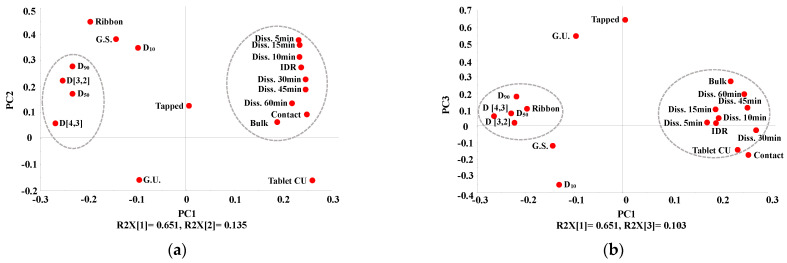
Score scatter plot of roller compaction: (**a**) PC1 and PC2, (**b**) PC1 and PC3. The group within the gray dotted lines located opposite each other indicate a negative correlation. Contact: contact angle; Diss.: dissolution: G.U.: granule uniformity: G.S.: granule strength; Tapped: tapped density; Bulk: bulk density; Ribbon: ribbon density; IDR: intrinsic dissolution rate.

Figure 7a shows the loading plot with PC1 and PC2 of roller compaction. According to Figure 7a, the dissolution, intrinsic dissolution rate, contact angle, bulk density, and tablet C.U. had positive loading values in PC1. In contrast, the granule size, ribbon density, granule strength, and granule uniformity, which are located on the other side, had negative loading values in PC1. The tapped density near zero indicates that it had a lower effect on PC1. In addition, granule size and dissolution, which are opposite to each other, had a negative correlation. In the case of PC2, granule uniformity and table C.U. had negative loading values and ribbon density, granule strength, granule size, dissolution, and intrinsic dissolution rate had positive loading values.

Figure 7b shows the loading plot with PC1 and PC3 of roller compaction. As is shown in Figure 7b, the granule uniformity and tapped density had positive loading values in PC3. Granule size D_10_, granule strength, contact angle, and tablet C.U., which are located on the other side had negative loading values. Granule size D(3,2), dissolution at 5, 10, and 30 min, and the intrinsic dissolution rate located near zero indicate that they had a lower effect on PC3. Additionally, the dissolution, bulk density, and intrinsic dissolution rate had a strong correlation because they aggregated. As granule size and ribbon density were aggregated, they had a strong correlation.

**Figure 8:**
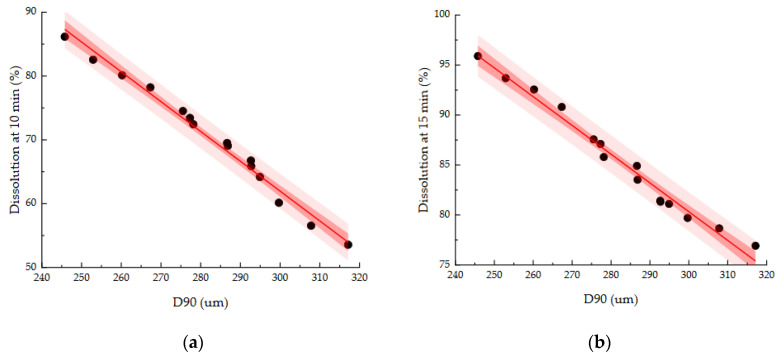
Fitted line plot of roller compaction process: (**a**) relationship between D_90_ and dissolution at 10 min, (**b**) relationship between D_90_ and dissolution at 15 min. The black point represents experimental data, the red line represents the best-fitted regression of data, the red-filled line is the 95% C.I. band, and the pink-filled line is the 95% P.I. band.

As was mentioned above, the dissolution in CQAs was used to establish the control strategy for the roller compaction process. Based on the Pearson correlation coefficient and PCA, we confirmed that granule size and dissolution profiles had a strong negative correlation in the roller compaction process. Figure 8 shows the fitted line plot of D_90_ versus dissolution at 10 min and D_90_ versus dissolution at 15 min. As is shown in Figure 8a, there was a negative correlation between D_90_ and dissolution at 10 min. In addition, the plot indicates a linear relationship with an R^2^ of 0.99, and an adjusted R^2^ of 0.98. The value of the residual sum of squares was 18.4, indicating that the model fit the data. In addition, as is shown in Figure 8b, D_90_ has a negative correlation with dissolution at 15 min. The plot shows a linear relationship with an R^2^ of 0.99, and an adjusted R^2^ of 0.98. The value of the residual sum of squares was 9.36, indicating that the model fit the data. Figure 8 shows that a decrease in granule size increases dissolution. This is because small granules have a larger surface area than large granules, so drug release is fast [24]. This means that monitoring IQAs in the roller compaction process can present target CQAs in real time, except for the drug product experiments after the process.

## 4. Conclusions

In this study, we established a control strategy for a combination drug prepared by the high-shear wet granulation and roller compaction processes using the integrated QbD approach with MVA. Response surface design was used to obtain the optimal process parameters of high-shear wet granulation and roller compaction. Based on the initial risk assessment, CPPs, IQAs, and CQAs were selected, and the effects of CPPs on IQAs and CQAs were confirmed by coded equations using ANOVA. After establishing the design space, we conducted Monte Carlo simulations to evaluate the risk of uncertainty in model predictions. The relationship between numerous variables presented in the QbD approach was confirmed through the Pearson correlation coefficient and PCA. The MVA results prove that the relationship between IQAs and CQAs can be a powerful tool to control and predict CQAs in high-shear wet granulation and roller compaction processes. In high-shear wet granulation process, IQAs such as granule size, bulk density, and intrinsic dissolution rate were significantly correlated with CQAs such as dissolution profiles, swelling properties, and contact angle. In particular, dissolution at 1 h had a strong correlation with the intrinsic dissolution rate and granule size, and dissolution at 3 h had a strong correlation with bulk density and granule size. In the roller compaction process, IQAs such as granule size, intrinsic dissolution rate, ribbon density, and granule strength were significantly correlated with CQAs such as dissolution profiles, tablet C.U., and contact angle. In particular, dissolution at 10 min and 15 min was strongly correlated with granule size. Based on these relationships, we can develop a control strategy for the production of high-quality drug products. The properties of the intermediate product can be controlled by adjusting the process parameters. Technologies such as PAT allow the real-time identification of the characteristics of intermediate products. The results of this study suggest that the combination of MVA and DoE in pharmaceutical process development can offer a deep understanding of the relationships among numerous variables, which is one of the most fundamental concepts for control strategy.

## Figures and Tables

**Table 1:** Quality target product profile (QTPP) for a bilayer tablet containing 10 mg dapagliflozin and 1000 mg metformin.

Quality Attributes		Target	Justification	Critical
Dosage form		Bilayer tablet with coating film	Pharmaceutical equivalence requirement: same dosage form.	Not critical
Dosage design		Bilayer tablet composed with immediate release and sustained release containing 10 mg of dapagliflozin and 1000 mg of metformin, respectively	Sustained release design needed to meet label claims.	Not critical
Route of administration		Oral	Pharmaceutical equivalence requirement: same route of administration.	Not critical
Dosage strength		dapagliflozin 10 mg/metformin 1000 mg	Pharmaceutical equivalence requirement: same strength.	Not critical
Pharmacokinetics		C_max_ within 2 h of dapagliflozin consumption under the fasting state and C_max_ withn 4.0 to 8.0 h of metformin consumption; bioequivalent to XIGDUO™ XR	Bioequivalence requirement.	Not critical
Stability		At accelerated conditions: 40 °C/75% RH At long term storage condition: 25 °C/60% RH	Equivalent to or better than the shelf-life of XIGDUO™ XR.	Not critical
Drug product quality attributes	Assay	90% to 110% *w/w* of label claim	Assay variability will affect safety and efficacy. Both material attributes and process parameters may affect the assay of the drug product. Thus, the assay should be evaluated throughout the product and process development.	Critical
	Content uniformity (C.U.)	Conforms to USP<905> uniformity of dosage units	Variability in content uniformity will affect safety and efficacy. Both formulation and process variables impact content uniformity, so this CQA should be evaluated throughout product and process development.	Critical
	Swelling property	Similar to XIGDUO™ XR	The swelling property, gel strength, weight gain, and mass loss of tablets may affect safety and efficacy as these are directly correlated with dissolution that will affect bioavailability.	Critical
	Weight gain			
	Mass loss			
	Gel strength			
	Dissolution	Metformin: 10% to 40% after 1 h; 40% to 70% after 3 h; more than 75% after 10 h. Dapagliflozin: more than 70% after 30 min	Failure to meet the dissolution specifications can affect bioavailability. Process variables affect the dissolution profile.	Critical
	Hardness	Ranging from 17.0 kp to 18.0 kp for metformin and from 27.0 kp to 28.0 kp for dapagliflozin	An extremely hard tablet could indicate excessive bonding potential between API and the excipients, which can prevent the proper dissolution of the tablet needed for accurate dosing.	Critical
	Friability	Not more than 1.0% *w*/*w*	Friability is a routine test as per the compendial requirements of tablets. A target of less than 1.0% *w*/*w* of mean weight loss assures a low impact on patient safety and efficacy and minimizes customer complaints.	Critical
	Contact angle	Similar to XIGDUO™ XR	The contact angle of tablets may affect safety and efficacy as it is directly correlated with dissolution that will affect bioavailability. Thus, it affects bioavailability. Thus, the contact angle should be evaluated throughout product and process development.	Critical
	Identification	Pharmaceutical equivalence requirement: Must meet the same applicable (quality) standards (i.e., identity, assay, purity, and quality).		Not critical
	Degradation products			Not critical
	Residual solvents			Not critical
	Microbial limits			Not critical
	Physical attributes			Not critical
Intermediate product quality attributes	Intrinsic dissolution rate	Similar to XIGDUO™ XR	Intrinsic dissolution rate and solubility are the main physicochemical aspects pertaining to drug absorption. The intrinsic dissolution test may offer greater correlation to the in vivo dissolution dynamic than the solubility test. Thus, the intrinsic dissolution test should be investigated throughout product and process development.	Critical
	Granule size		The particle size affects flowability and content uniformity. Thus, size should be evaluated throughout product development.	Critical
	Granule flowability		Granule flowability is relative to granule size, assay, CU, and dissolution. Thus, granule flowability should be evaluated throughout the product and process development.	Critical
Container closure system		Container closure system qualified as suitable for this drug product	Needed to achieve the target shelf-life and to ensure tablet integrity during shipping.	Not critical
